# Optimization of the Green Conventional Extraction Method of Sericin from Silkworm

**DOI:** 10.3390/polym17131823

**Published:** 2025-06-30

**Authors:** Daniel Stiven Burgos Gomez, Maite Rada-Mendoza, Diana M. Chito-Trujillo

**Affiliations:** G.I. Biotecnología, Calidad Medioambiental y Seguridad Agroalimentaria (BICAMSA), Departamento de Química, Facultad de Ciencias Naturales, Exactas y de la Educación, Universidad del Cauca, Campus Universitario de Tulcán, Carrera 2 # 15N Esquina, Popayán 190002, Colombia; danielbg@unicauca.edu.co (D.S.B.G.); dchito@unicauca.edu.co (D.M.C.-T.)

**Keywords:** silkworm (*Bombyx mori*), silk proteins, sericin, conventional extraction, optimization, green extraction

## Abstract

In the silk production process, cocoons from *Bombyx mori* worm are degummed and separated from their components. This step generates large residual quantities of an aqueous solution containing various chemical substances, including sericin—a protein that, when discarded improperly, negatively impacts the environment. Sodium bicarbonate and coconut soap are commonly used in the degumming process. The phosphates in the soap and the sodium bicarbonate increase the biological oxygen demand (BOD) and chemical oxygen demand (COD), leading to water contamination. In this study, a Box–Behnken experimental design was used to maximize the extraction of sericin through a conventional extraction under chemical-free conditions. From a total of 45 experiments, the optimal extraction conditions were identified as a solid-to-liquid ratio of 1:20 *w*/*v*, a temperature of 120 °C, and 90 min of extraction time. Sericin yields ranged from 9% to 18%. Infrared spectroscopic characterization of the extracted sericin confirmed the presence of protein-specific functional groups and common interactions associated with β-sheet structures. Fractions of high molecular weight (50 kDa to 200 kDa), identified by means of Sodium dodecyl sulfate–polyacrylamide gel electrophoresis (SDS-PAGE) analysis, demonstrate the potential functionality of extracted sericin for the development of biopolymer films useful in biomedical and food industry applications. The optimized methodology is a good alternative to recycle the waste of sericulture chain for obtaining extracts enriched in sericin, as well as to promote the mechanization of artisanal production processes.

## 1. Introduction

Silk is a natural fiber produced by various arthropods, including spiders, scorpions, and certain insects such as bees and silkworms. Among these, *Bombyx mori*—a species of the Bombycidae family—is the most commercially important due to its ease of domestication and favorable conditions for silk harvesting, in contrast to wild silks from Saturniidae or Lasiocampidae families, which require more complex extraction processes [1]. The domestication and breeding of Bombyx mori, which feeds on mulberry leaves and produces cocoons used for silk extraction, is known as sericulture [2]. This process aims to obtain high-quality cocoons intended for use in textile manufacturing. Silk is valued for its bright, soft, and strong fibers, and has historically been regarded as a high-quality natural textile material. The silkworm, a holometabolous insect of the order Lepidoptera [3], produces silk composed mainly of two proteins: fibroin (65–85%) and sericin (15–35%) [4].

Fibroin is a crystalline, water-insoluble structural protein, while sericin (SER) is a hydrophilic, globular protein that surrounds the fibroin and acts as a natural adhesive, maintaining the integrity of the silk fiber [5]. SER is composed of 18 amino acids, with particularly high proportions of serine (32%), aspartic acid (18%), and glycine (16%) [6]. It contains 70 repeated sequences of 38 amino acids, rich in hydroxylated and polar residues (45.8% and 42.3%, respectively), resulting in a molecular weight ranging from 20 to 400 kDa [5,7,8].

The degumming process, used to extract SER, renders silk fibers soft and lustrous. Sericin is a major by-product in the textile industry, with an estimated 50,000 tons produced annually [9]. However, it is commonly discharged into wastewater, contributing to increased Chemical Oxygen Demand (COD) and Biological Oxygen Demand (BOD), raising environmental concerns [10]. Recovering sericin represents both an ecological and economic opportunity.

Due to its biodegradability and biocompatibility, SER is gaining increasing attention in food, cosmetic, and biomedical fields. It exhibits ultraviolet (UV) resistance, antioxidant, antibacterial, and gelling properties. Its polar amino acid side chains enable easy cross-linking and function as effective wetting agents [11]. Additionally, SER has demonstrated immunological inertness; minimal inflammatory responses and favorable antioxidant activity in animal tissues have been reported [5,12,13,14,15].

Traditional extraction methods using hot water solubilize sericin without the use of chemical agents while preserving its bioactive properties. However, inadequate control of temperature and time can result in thermal degradation [16]. Emerging techniques—such as microwave-assisted extraction, steam treatment, supercritical CO_2_, and ultrasonication—are more sustainable alternatives but remain technically complex and challenging to scale for small sericulture enterprises [16].

This study proposes a simple physical extraction method using only heat and distilled water, completely avoiding the use of chemical agents. It aims to demonstrate that sericin can be efficiently extracted through accessible and low-cost means, promoting added value in sericulture microenterprises. Although similar approaches have been reported by Chirila et al. (2016) and Bascou R. et al. (2022) [17,18], their methodologies lack a robust experimental design. In contrast, Sothornvit et al. (2010) [19] implemented statistical analysis but did not account for key variables such as the solid-to-liquid ratio. The present study addresses these gaps by introducing a complete experimental design that includes all critical factors influencing extraction efficiency.

## 2. Materials and Methods

### 2.1. Sample Preparation

**Figure 1:**
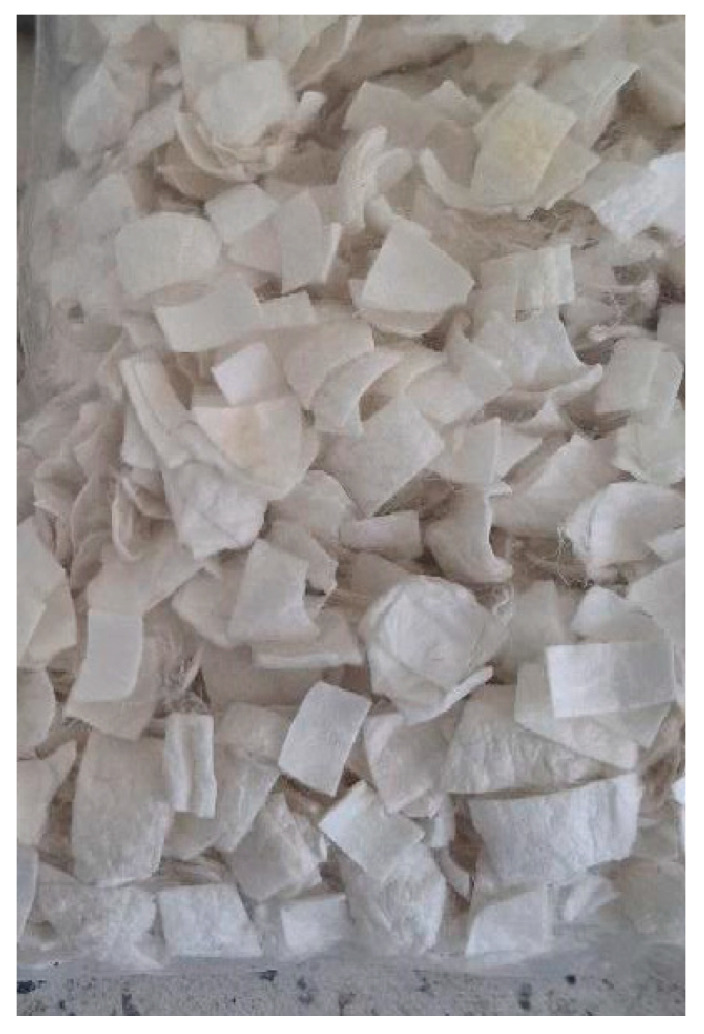
Cocoons cut into 1 cm^2^ squares.

Cocoons of the silkworm *Bombyx mori* supplied by the Association COLTESEDA of the municipality of Timbio in the department of Cauca-Colombia (Timbio-Cauca, Colombia) were used. Cocoons were open and without larvae inside, and prior to the extraction, these were visually selected according to their appearance to attain cocoons that did not contain any dirt (larval waste product) to obtain extracts of high purity. Then, the cocoons were cut into small squares of approximately 1 cm^2^ as shown in Figure 1.

### 2.2. Conventional Sericin Extraction

Degumming was performed by adding a specific number of cocoons to a defined volume of water, as determined by the experimental design (Table 1) for each extraction. Each experiment was conducted at a fixed temperature and duration using a conventional reflux system to prevent water evaporation.

After the extraction process, the skein (resulting solid, fibroin) must be removed by vacuum filtration; subsequently, this skein was dried at a temperature of 105 °C for 2 h in a Thermo Scientific Precision 658 (Waltham, MA, USA) oven and then it was allowed to cool to room temperature and weighed for application of Equation (1):

(1) $$Extraction\;(\%)=\frac{Initial\;cocoon\;weight-Dry\;skein\;weight}{Initial\;cocoon\;weight}$$

### 2.3. Experimental Design

A Box–Behnken design was applied to evaluate the effect of three independent variables, as reported by Castrillón (2017) [20]: cocoon:water ratio, temperature (°C), and time (min) at experimental ranges, as shown in Table 1. A total of 15 experiments (three-level design with a subset of runs in the full three-level factorial and three center points per block to estimate the experimental error) were performed in randomized order (see Table 2).

The factor levels used in this study were determined based on a literature review and previous experimental work carried out by the authors. This is a quadratic design, capable of handling between three and five factors [20], for each of which there are three levels: low, medium, and high (Table 1). The experimental data were fitted to a quadratic model by regression. This design allows the identification of the interactions and main effects of the variables, as well as the quadratic term which indicates the curvature of the response surface [21]. A total of 15 combinations of factors and levels were obtained, combinations that were performed in triplicate, resulting in a total of 45 experiments (Table 2); the design was randomized in order to avoid statistical bias [22].

The Minitab^®^ Statistical Software version 18 was used to perform the statistical analyses, and to generate the experimental design.

### 2.4. Structural Analysis by Fourier Transform Infrared Spectroscopy (FTIR)

The identification of functional groups associated with the protein structure and structural analysis, presence of functional groups and configuration of the extracted sericin was performed using infrared spectroscopy with a Thermo Scientific Nicolet iS10 FT-IR spectrometer (Waltham, MA, USA).

### 2.5. Molecular Weight Distribution (SDS-PAGE)

The sericin sample, once lyophilized, was dissolved in buffered saline solution at pH 7.4 (11.9 mM Phosphates, 137 mM NaCl, and 2.7 mM KCl; Fisher BioReagents^®^ (Fair Lawn, NJ, USA)). A volume of 500 µL of buffer was used; however, incomplete dissolution of the sample was observed. To improve solubility, dimethyl sulfoxide (DMSO) 4% to 20% was added.

Protein analysis was performed using an SDS-PAGE-Tricine (Fair Lawn, NJ, USA) polyacrylamide gel (12.5% resolving gel/5% stacking gel). The sample was treated with a loading buffer containing β-mercaptoethanol and heated at 100 °C for 10 min, to induce protein denaturation. It was then centrifuged at 10,000 rpm for 1 min. In the wells of the gel, a volume of 15 µL of the treated sample was mixed with 5 µL of loading buffer (protein concentration previously determined with a NanoDrop 1000 spectrophotometer, Thermo Scientific (Waltham, MA, USA), was loaded into the gel wells. Electrophoresis was run at 100 V for 120 min followed by staining with Coomassie Brilliant Blue R-250 (Levinstein Ltd., Kumasi, Ghana) for at least 4 h.

Finally, the gels were incubated in deinking solution until the bands were clearly visualized. A Precision Plus Protein™ Dual Xtra Prestained Protein Standard marker (BioRad^®^ Hercules, CA, USA) was used and treated under the same conditions.

## 3. Results and Discussion

### 3.1. Optimization of the Conventional Method Without Chemicals

The ratio, temperature, and extraction time were selected as the most relevant parameters in the optimization of a conventional method for the efficient extraction of sericin from silkworm cocoons. These cocoons are composed of two primary proteins: fibroin and sericin. This compositional characteristic supported the optimization of the simple, mechanical extraction method without the use of chemicals agents assayed in this study. 

Notably, sericin contains several hydrophilic (water-loving) amino acids in its structure, such as serine, threonine, aspartic acid, asparagine, glutamic acid, and glutamine [23], whereas fibroin is primarily composed of hydrophobic amino acids, including glycine and alanine [24].

Hence, hot water separation emerges as an ideal methodology for the treatment of this type of matrix and its implementation is feasible within production systems where process quality and cost-efficiency remain areas for improvement.

The ratio between the cocoons and the volume of water used for extraction, along with temperature and the time of the process, are variables that are of great importance for the study. Establishing an appropriate proportion between the mass of the cocoons and the volume of water ensures an efficient sericin solubilization without saturating the solution or wasting resources. Temperature is a key factor for the extraction of sericin. At elevated temperatures, the β-sheet structures of sericin begin to degrade, increasing its solubility in hot water. For instance, it has been observed that the third sericin layer is more easily removed in water at 83 °C, whereas certain insoluble fractions require temperatures up to 100 °C for complete extraction. Conversely, temperatures below 80 °C result in poor protein solubility (as confirmed by tests conducted by the authors). The temperature range used in this research allows breaking the protein matrix without complete degradation, allowing for controlled denaturation. However, excessive temperatures can lead to uncontrolled degradation, negatively affecting extraction quality.

According to Murillo-Usuga et al. (2022), time is statistically significant in the extraction process, since the time to which the cocoon is subjected to heating is crucial to obtain good extractions [25]. Table 2 gathers the average of the percentage of extraction determined under the different extraction conditions evaluated.

The coefficients of variation were less than 5% and given that a 95% confidence level was applied in this research, it can be concluded that all the data obtained for each experiment could be averaged and reported. Extraction percentages ranging from 9.05% to 17.77% were attained being a wide range due to the way in which the starting material (silkworm cocoons) can interact with the different changes in the independent variables of the experimental design. On the other hand, the values of SER extraction percentages reported in the cocoon are 15–35% [4].

An analysis of variance (ANOVA) was then carried out and the results are summarized in Table 3. The data explain the statistical significance of the above model; its individual and interaction having a *p*-value of 0.000 for the model demonstrates a statistically significant fit to the model, since at least one of the terms considered has a significant effect on yield [26,27].

Analysis of the linear effects revealed that the ratio variable did not have a statistically significant impact on the extraction percentage (*p* = 0.17), whereas the temperature and time did show significant effects (*p* = 0.00). However, even when a term is not individually significant, its coefficient may still influence the position of the optimal point, particularly through interactions with other variables. Therefore, the model may suggest an optimal value for the “Ratio” factor, which, despite its isolated effect, may not be statistically strong [28].

Now, the quadratic effects are analyzed, and it was found that none of the quadratic interactions of the variables present significance in terms of extraction yield, suggesting that there is no presence of optimal points in a quadratic model and confirming that the model is linear [29]. In the case of second-order interactions, *p*-values > 0.05 were recorded for all types of interactions, except for the temperature–time interaction (*p* = 0.06), which is close to the significance threshold and would significantly influence the response.

The model yielded an R^2^ equal to 76.37%, this is to say the percentage indicates that 76.37% of the variability of the extraction percentage is explained by the fitted model. Thus, the model captures much of the variability in the data [30]. The diminished adjusted R^2^ of the model (68.49%) is correlated to those quadratic terms that do not contribute significantly to the response. On the other hand, the predictive R^2^ coefficient is a fundamental metric in the validation of regression models, since this coefficient evaluates the capacity of the model to predict the results of variables that were not used In the adjustment [31]. The relatively lower predictive R^2^ (53.73%) suggests a substantial variability in the test data that is not explained by the model [31], but could be an inherent variability to the SER extraction process that has not been considered by the independent variables evaluated in this research. For instance, not all cocoons have the same SER content, and this fact may experimentally affect the extraction percentages and induce the mentioned variability inherent to the extraction process.

**Figure 2:**
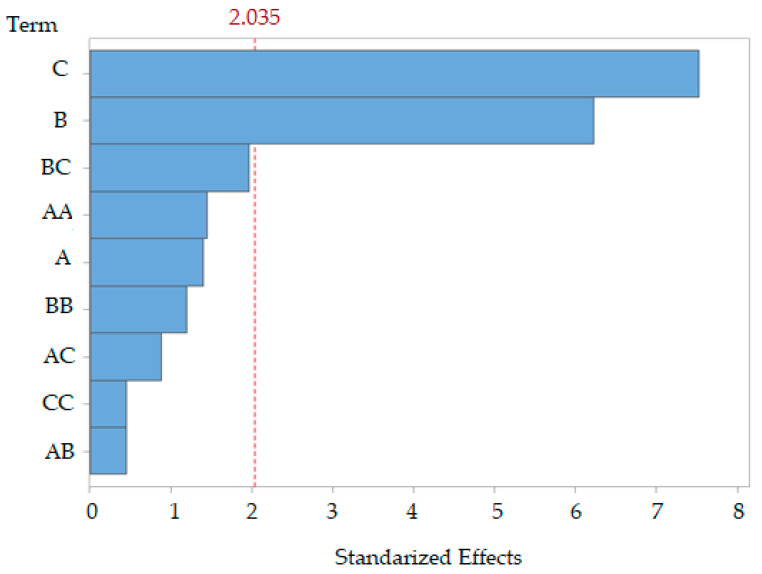
Pareto chart of normalized (*p* = 0.05) linear, quadratic, and interactive effects of % SER extraction. A: Ratio, B: Temperature, and C: Time.

The pareto diagram shown in Figure 2 confirms the previous analysis, where factors C and B are above the standardized effect value of 2.035, which means that these are the variables that have statistical significance in the model proposed. On the other hand, the BC interaction is close to this limit of significance, so it may have some influence on the response of the model.

**Figure 3:**
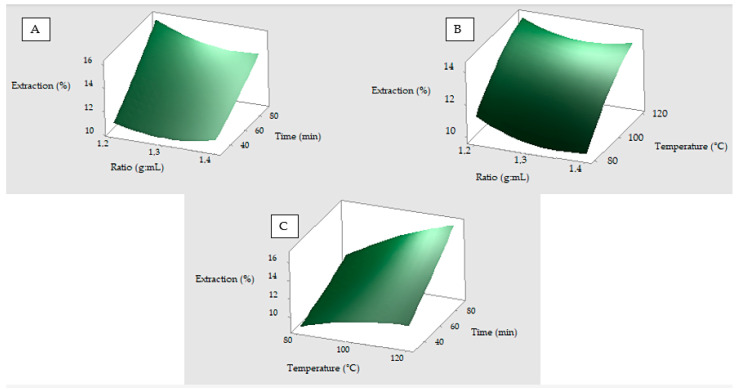
Response surface plots showing the effects of variables on extraction of SER. (**A**) The interaction of ratio and temperature. (**B**) The interaction of ratio and time. (**C**) The interaction of temperature and time. Experimental conditions: ratio 1:30 (g:mL), temperature 100 °C, and time 60 min. In (**A**,**B**), green dark color depicts the area with the highest response while this is depicted by a green clear color in (**C**).

The response surface plots depict the combined effect of two factors on the response variable. This analysis is essential to identify the optimal regions of operation and to understand the interaction between the factors. Figure 3A shows an increase in the response variable as the temperature increases. In Figure 3B, a positive interaction for the time factor is observed, while the ratio factor continues to present a negative effect on the response since high values decrease the yield. The best performance between is expected at low values of ratio and prolonged times.

Otherwise, the percentage extraction increases with increasing temperature and time, see Figure 3C; the surface shows an upward slope without marked curvature, suggesting that both factors have a positive linear effect on the response variable. No curvature is observed in any of the response surface plots, indicating that this model, as previously analyzed, is of the linear type.

The Variance Inflation Factor (VIF) evaluates multicollinearity. When the VIF of a variable is greater than 5 or 10, it is considered that multicollinearity exists [32], in which case, as shown in Table 3, there are no collinearity problems in the factors used in the design, as all values are equal to 1.

The experiment conditions corresponded to a 1:20 ratio, temperature of 100 °C, and extraction time of 90 min (as shown in Table 2) yielded the highest average sericin extraction percentage. This value represents the percentage by weight lost from the cocoon due to sericin removal.

According to the literature, sericin accounts for approximately 15–30% of the total cocoon weight [4]. For the purpose of this study, an average sericin content of 22.5% was assumed to estimate extraction efficiency. Thus, an extraction yield of 22.5% is considered to represent 100% efficiency. In our case, a sericin extraction yield of 17.32% corresponds to an efficiency of 76.97%.

In a previous study, H. Yun et al. (2013) sericin extractions were conducted using 8M Urea and a mixture of Urea–mercaptoethanol 5% (*v*/*v*), both at 80 °C for 10 min, as well as using NaCl at 25 °C for 15 h, resulting in extraction percentages values of 3.7, 9.0, and 9.0%, respectively. In addition, one assay under basic conditions commonly by small-scale producers—100 °C for 1 h with 0.2 M Na_2_CO_3_—which yielded an extraction percentage of 13.2%, equivalent to an efficiency of 67.5%. A final test conducted using only water at 120 °C for 1 h produced a sericin yield of 8.3%, corresponding to an efficiency of 42.7% [33]. Bascou, R. et al. (2022) carried out a conventional extraction using hot water with a 1:50 ratio, at 100 °C for 30 min. However, in the absence of proper optimization, an extraction efficiency of 65.2% was reported [17]. These findings support the omission of chemical agents or basic media in the extraction process as extraction alternatives, demonstrating that satisfactory yields can be achieved through an appropriate optimization of key process parameters. The results of this study highlight that optimizing factors such as ratio, temperature, and time can significantly enhance both yield and efficiency of sericin extraction.

**Figure 4:**
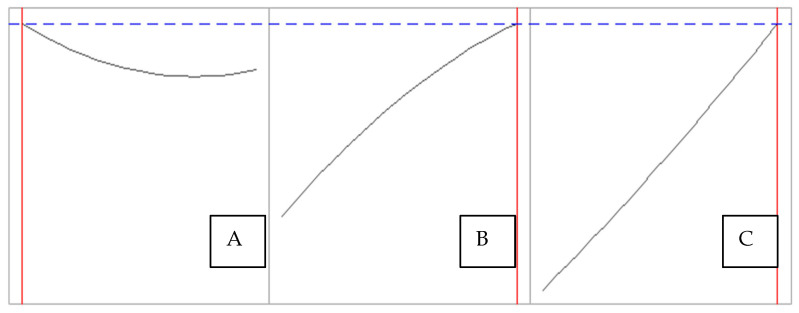
Partial least squares (PLS) prediction profiler of each variable and desirability function with extrapolation control for the optimization of sericin extraction. (**A**) Ratio (1:49 to 1:20), (**B**) temperature (126.8 °C to 120.0 °C), (**C**) time (204.8 min to 180.0 min). The maximum predicted response is 17.8705% with a desirability of 1.00. The black line depicts the behavior of each variable in order to determine its optimum point. The blue line represents the expected maximum. Red lines delimit the values evaluated at the upper and lower limits.

As shown in Figure 4, the optimal extraction conditions are: ratio, 1:20; temperature, 120 °C; and time, 90 min. Under these conditions, the model predicts that an extraction percentage of 17.87% can be achieved and presents a global desirability level of 1.00. This function is a common technique in multiresponse optimization [34]; in this case, it indicates that the maximum predicted response is within the experimental space and satisfies the factorial conditions. For the experimental application of these conditions, three extractions were performed and an average result of 18.51% was found with a CV of 2.22%. This means that although the value predicted by the model was not obtained exactly, the performance increased with optimized conditions. One of the reasons for this is because the model is mathematical and theoretical, which does not consider the experimental conditions that may occur in the laboratory. With the optimized conditions, an extraction percentage of 82.28% was achieved.

It is important to note that while the optimized model suggests that increasing factors such as temperature or time might further enhance extraction, it is based on a mathematical and statistical approach that does not account for protein stability. If sericin is exposed to high temperatures for extended periods, it may undergo thermal hydrolysis, leading to the alteration of its functional properties. Exceeding the temperature of 120 °C beyond the optimized extraction time may promote and accelerate peptide bond cleavage, resulting in protein degradation.

### 3.2. Sericin Characterization

**Figure 5:**
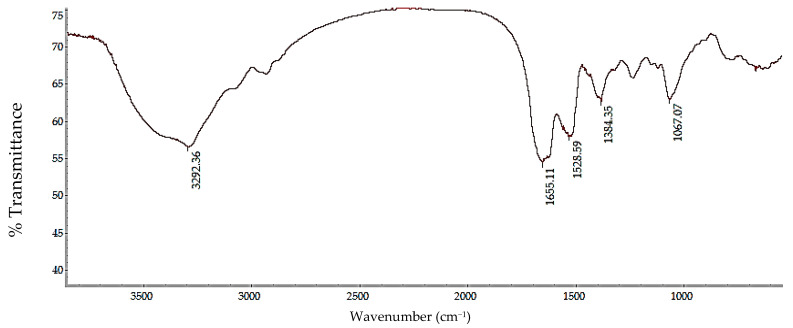
FTIR of sericin extracted from silkworm cocoons, dehydrated by lyophilization.

Figure 5 shows the infrared spectrum obtained on the lyophilized sample resulting from the experiment associated with the highest yield, which is the experiment with the optimized conditions (ratio, 1:20; temperature, 120 °C; and time, 90 min). The spectrum reveals the presence of band vibrations characteristic of the amide groups in the protein can be evidenced: for amide A and B (3292.36 cm^−1^), this peak is attributed to the stretching of the N-H bonds which overlap with the hydroxylated amino acid residues (OH), such as serine and threonine [35]. Amine I (1655.11 cm^−1^) represents the stretching of C=O bonds, which are involved with the main chain of polypeptides, and therefore, they are more sensitive to the secondary structure and molecular orientation of the protein; Amide II (1528.59 cm^−1^) is associated with the stretching of C-N bonds and the deformation of N-H bonds; and finally, Amide III (1384.35 cm^−1^) is associated with the same bonds as Amide II [36]. With the peaks, it could be evidenced that the sample presents main peaks belonging to a β-sheet structure [37]. Kim et al. (2012) presented similar results, also observing that to achieve a change of structure to random spiral, lyoprotectants can be added, which could favor the solubility of the sample [37]. The β-sheet structures identified in sericin during this study have been extensively explored in the development of biomedical materials, including scaffolds for tissue regeneration, drug delivery systems, and soft tissue repair materials. Their ability to form stable and biocompatible conformations makes them highly suitable for these applications [38].

Furthermore, studies have shown that sericin with β-sheet structures is effective in promoting the nucleation of hydroxyapatite (HAP) crystals, a major component of bone. In the presence of Ca^2+^ ions, sericin undergoes a conformational change from a random coil to a β-sheet structure, facilitating its self-assembly into a nanofibrous network that serves as a template for the oriented growth of HAP crystals. This property is particularly valuable in the design of biomaterials for bone repair and regeneration [39].

**Figure 6:**
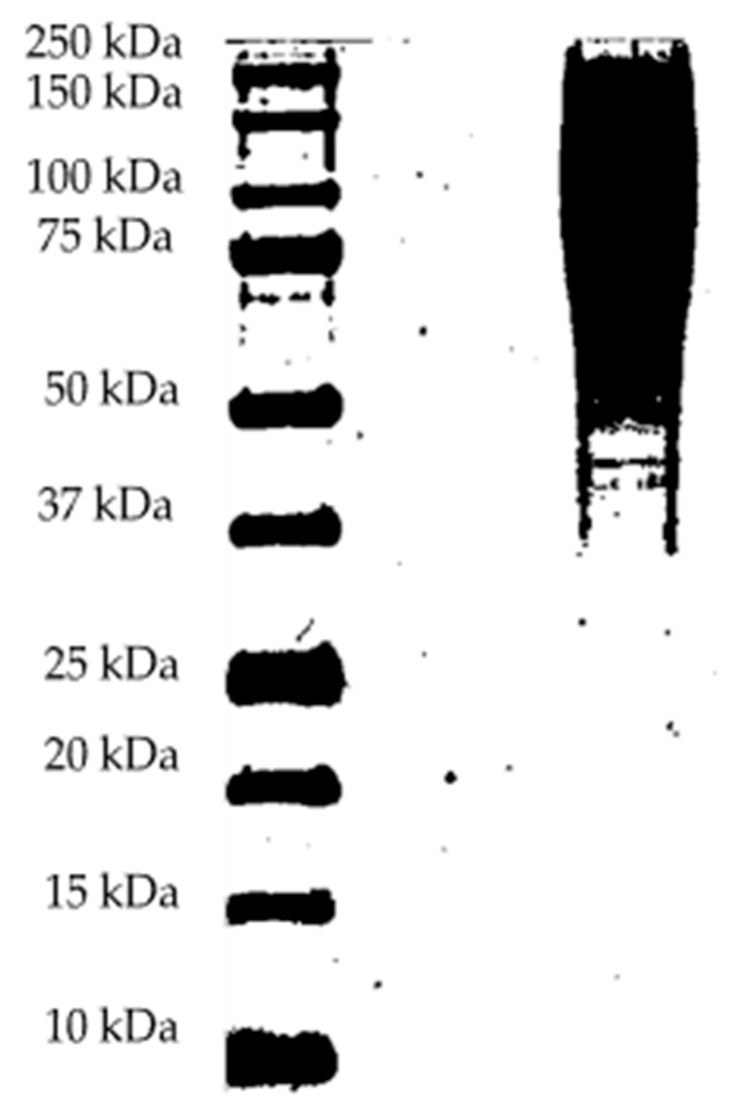
Sodium dodecyl sulfate–polyacrylamide gel electrophoresis (SDS-PAGE) analysis. The first well contains the molecular weight marker, and the second well contains the sericin sample extracted under the optimized conditions of this study.

Figure 6 shows the results of the SDS-PAGE (Tyrosine staining) analysis used to determine the molecular weight of the sericin extracted under the optimized conditions of this study. A broad, smear-like band was observed, ranging from approximately 50 kDa to 200 kDa, along with a well-defined band near 40 kDa. The presence of the smear indicates a distribution of high-molecular-weight fractions, which is commonly observed when proteins are not deeply hydrolyzed.

Previous studies have shown that chemical degumming promotes protein hydrolysis, resulting in lower molecular weights [17]. In contrast, hot water extraction preserves high-molecular-weight fractions by avoiding chemical-induced degradation. This explains the diffuse band at higher molecular weights in Figure 6, which likely results from the presence of multiple sericin fractions with similar molecular weights that could not be resolved with greater definition.

Our findings are consistent with those reported by Bascou et al. (2022) [17], who also observed a diffuse band at high molecular weights (200 kDa). However, a key difference is that their analysis revealed a defined band at 24 kDa, whereas our study identified a well-defined band between 37 kDa and 50 kDa, centered around 40 kDa. This difference may be attributed to the controlled extraction conditions used in our study, which help preserve the structural integrity of sericin.

On the other hand, authors such as Kunz, R. et al. (2016) have found sericin molecular weights between 20 and 400 kDa [5]; Gulrajani et al. (2009) appreciated bands at 25, 66, and 90 kDa, and a broad smear band at 205 kDa in the analysis of sericin extracted by application of high pressure and high temperatures [40].

Sericin has been investigated for the development of biopolymer films for biomedical and food industry applications. Due to its organic nature and film-forming capacity, sericin-based materials may be used to coat foods, preventing damage from moisture and oxygen. These applications are typically required.

The sericin extraction method based on water and reflux heating is a widely used, environmentally friendly strategy, due to its simplicity and low ecological impact. However, it presents significant limitations that compromise its effectiveness in applications requiring high-purity proteins or preservation of specific functional properties. Although the method promotes partial denaturation of sericin at temperatures between 80 °C and 120 °C—facilitating its solubilization—prolonged exposure or excessive temperatures may accelerate peptide bond cleavage and disrupt secondary structures such as β-sheets, which are essential for many of sericin’s bioactive properties [11].

This degradation leads to a reduction in the protein’s antioxidant, anti-inflammatory, and regenerative activities. Moreover, the use of pure water limits control over pH conditions, which can increase undesired hydrolysis and reduce process reproducibility. From an industrial perspective, the requirement for large volumes of water and the high energy consumption associated with reflux heating constrain the economic scalability of this method, particularly when high-molecular-weight sericin is needed for biomedical or pharmaceutical applications [17].

Despite the limitations of the statistical model and its possible variability, the extraction technique assayed remains suitable for textile and cosmetic applications, where greater molecular heterogeneity is acceptable and environmental sustainability and energy efficiency are a priority [41]. For more demanding applications, it is advisable to combine this technique with purification processes. Biomedical or food applications must meet certain quality standards regarding purity, reproducibility and good manufacturing practices (GMP) [42]. Thus, an ultrafiltration process subsequent to the SER extraction would ensure the quality and structural uniformity of the extracted proteins [42].

In terms of scalability, integrating renewable energy sources to power the extraction system (such as solar energy)—along with the implementation of water recirculation mechanisms—would enhance the overall sustainability and efficiency of the reflux-based extraction process in industrial environments with ecological approach [43].

## 4. Conclusions

In this study, the conventional extraction of sericin without the use of chemicals rather than water from silkworm cocoons was optimized using a Box–Behnken design. The predictive model developed was significant (*p* < 0.05) with an R^2^ of 76.37%, indicating a good predictive ability. Temperature and time were found to significantly influence the extraction, while ratio had no relevant impact. Optimal conditions (ratio, 1:20; temperature, 120 °C; time 90 min) allowed reaching an experimental yield of 18.51%, which is higher than previous studies employing chemical agents such as Na_2_CO_3_.

In addition, characterization by infrared spectroscopy and SDS-PAGE electrophoresis confirmed the integrity of the sericin extracted. These results show that the optimized hot water process is more efficient, sustainable, and economically viable than conventional chemical agent methods and the preservation of high-molecular-weight fractions, being functional for the development of biopolymer films for biomedical and food industry applications. These results show that the optimized hot water process is more efficient, sustainable, and economically advantageous than conventional chemical agent methods.

## Figures and Tables

**Table 1:** Ranges of the three independent variables of the Box–Behnken design.

Ratio (Cocoon:Water)	Temperature (°C)	Time (min)
1:20	80	30 *
1:30 *	100	60
1:40	120 *	90

* Conditions reported by Castrillón, 2017 [20].

**Table 2:** Box–Behnken experimental design for optimization of conventional extraction of sericin from silkworm cocoons. Percentage of extraction yielding and variation coefficients (VCs).

Ratio (Cocoon:Water) g:mL	Temperature (°C)	Time (min)	Average Extraction (%)
1:20	100	30	9.20 VC: 1.49
1:40	100	30	9.52 VC: 3.33
1:30	80	30	10,05 VC: 4.74
1:30	120	30	12.59 VC: 2.53
1:20	80	60	11.65 VC: 3.24
1:40	80	60	10.08 VC: 0.20
1:20	120	60	14.27 VC: 3.60
1:40	120	60	13.44 VC: 2.88
1:30	100	60	12.28 VC: 2.36
1:30	100	60	12.35 VC: 2.78
1:30	100	60	12.13 VC: 3.44
1:30	120	90	15.43 VC: 4.76
1:30	80	90	9.69 VC: 4.54
1:20	100	90	17.32 VC:4.04
1:40	100	90	16.20 VC:1.73

**Table 3:** Analysis of variance using a non-linear regression model for the values of % extraction of SER extracted from silkworm cocoons.

Variable	Sum of Squares	Freedom Degrees	Value F	Value *p*	VIF
Block	0.30	2	0.08	0.93	-
Ratio	3.86	1	1.96	0.17	1.00
Temperature	76.40	1	38.84	0.00	1.00
Time	111.63	1	56.75	0.00	1.00
Rate^2^	4.15	1	2.11	0.16	1.01
Temperature^2^	2.84	1	1.44	0.24	1.01
Time^2^	0.41	1	0.21	0.65	1.01
Ratio × Temperature	0.41	1	0.21	0.65	1.00
Ratio × Time	1.54	1	0.78	0.38	1.00
Temperature × Time	7.66	1	3.89	0.06	1.00
Model	209.76	11	9.69	0.00	-
Missing adjustment	64.11	27	17.83	0.00	-
Error	0.80	6	-	-	-
Total	274.67	44	-	-	-
R^2^	76.37%				
R^2^-Adjusted	68.49%				
R^2^-Predictive	52.73%				

## Data Availability

The original contributions presented in this study are included in the article. Further inquiries can be directed to the corresponding author.
